# Mandible histology in *Metoposaurus krasiejowensis* (Temnospondyli, Stereospondyli) from the Upper Triassic of Poland

**DOI:** 10.7717/peerj.12218

**Published:** 2021-09-30

**Authors:** Kamil Gruntmejer, Adam Bodzioch, Dorota Konietzko-Meier

**Affiliations:** 1 European Centre of Palaeontology, University of Opole, Opole, Poland; 2 Institute of Biology, Laboratory of Palaeobiology, University of Opole, Opole, Poland; 3 Institute of Geosciences, Division of Paleontology, University of Bonn, Bonn, Germany

**Keywords:** Temnospondyli, Late Triassic, Histology, Lower jaw, Feeding, Dermal bone

## Abstract

Recent studies that have systematically augmented our knowledge of dermal bones of the Late Triassic temnospondyl amphibian *Metoposaurus krasiejowensis* have mostly focused on shoulder girdle elements and the skull. So far, histological data on the mandible are still scant. For the present study, two mandibles have been examined, using 50 standard thin sections. Dermal bones of the mandible reveal a uniform diploë structure, with the external cortex consisting of moderately vascularised, parallel-fibred bone, as well as a distinct alternation of thick zones and thinner annuli. Dense bundles of well-mineralised Sharpey’s fibres are seen in the external cortex over the entire length of the mandible. The trabecular middle region is highly porous and well vascularised, showing small primary vascular canals and more numerous secondary osteons; irregular erosion spaces occur in large numbers as well. The thin and poorly vascular internal cortex consists of parallel-fibred bone. The articular is not a dermal bone in origin, having been formed of a thin layer of avascular cortex and a very extensive, trabecular middle region. In contrast to the dermal bones of the mandible, the articular developed from a cartilaginous precursor, as evidenced by numerous remains of calcified cartilage in the central parts of the bone. Histological variability is extremely high along the mandible, its anterior part being characterised by high compactness and biomechanically good resistance in contrast to the highly porous posterior parts. Distinct variations of bone thickness and degree of bone porosity in specific areas of the mandible, may be due to local differences in biomechanics during feeding. The microstructure of the mandible corroborates a previous study of the active and ambush predation strategy in metoposaurids.

## Introduction

Temnospondyli constituted a large clade of extinct amphibians which first appeared during the Early Carboniferous. In the Permian and Triassic, they achieved their greatest radiation and reached peak diversity; the clade became extinct in the Early Cretaceous (Milner, 1990). Metoposaurids were cosmopolitan temnospondyls in freshwater ecosystems during the Late Triassic (Carnian-Norian) (Schoch & Milner, 2000; Schoch, 2013). Skeletal remains of these large amphibians are known from North America (Hunt, 1993; Lucas, Spielmann & Hunt, 2007; Lucas, 2015; Gee, Parker & Marsh, 2017), Africa and Madagascar (Dutuit, 1976, 1978; Hunt, 1993), India (Chowdhury, 1965; Sengupta, 1992, 2002) and western and central Europe (Von Meyer, 1842; Schoch & Milner, 2000; Sulej, 2002, 2007; Brusatte et al., 2015). Characteristic features of their anatomy included a dorso-ventrally flattened body, short limbs and huge parabolic skulls with anterolaterally positioned orbits (*e.g*., Schoch & Milner, 2000). Since publication of the first paper on the genus *Metoposaurus* (Von Meyer, 1842), our knowledge of its ecology has consistently grown (see *e.g*., Ochev, 1966; Howie, 1970; Hunt, 1993; Dzik et al., 2000; Sulej, 2007; Konietzko-Meier & Sander, 2013; Konietzko-Meier, Bodzioch & Sander, 2013; Fortuny, Marcé-Nogué & Konietzko-Meier, 2017). *Metoposaurus krasiejowensis* Sulej, 2002 is now the best-known temnospondyl, following studies of its osteology (Barycka, 2007; Dzik & Sulej, 2007; Sulej, 2007; Antczak & Bodzioch, 2018), histology (Konietzko-Meier & Sander, 2013; Gruntmejer, Konietzko-Meier & Bodzioch, 2016; Teschner, Sander & Konietzko-Meier, 2018) and functional biomechanics (Fortuny, Marcé-Nogué & Konietzko-Meier, 2017; Konietzko-Meier et al., 2018; Gruntmejer, 2012; Gruntmejer et al., 2019a, 2019b).

Recent histological studies on metoposaurids have focused mainly on characteristics of long bones such as femora (Konietzko-Meier & Sander, 2013) and humeri (Teschner, Sander & Konietzko-Meier, 2018; Teschner et al., 2020) and ribs (Gądek, 2012), as well as vertebrae (Konietzko-Meier, Bodzioch & Sander, 2013; Konietzko-Meier, Danto & Gądek, 2014; Danto, Witzmann & Fröbisch, 2016; Gee, Parker & Marsh, 2017).

The histology of dermal bones in temnospondyls is still poorly known. The first description was provided by Gross (1934) for *Mastodonsaurus*, *Metoposaurus* and *Plagiosternum*. More detailed characteristics of dermal bones were presented decades later for a wider group of early tetrapods (Witzmann, 2009; de Buffrénil et al., 2016). Based on these studies it was concluded that dermal bones represented a metaplastic origin and exhibited a diploë structure with a well-differentiated external cortex, middle region and internal cortex. Witzmann (2009) also provided a short description of thin sections of a few dermal bone fragments of *Metoposaurus*. However, it is not clear if the bones examined belonged to the skull or the pectoral girdle (Witzmann, 2009). Gądek (2012) presented the first histological description of the clavicle of *Metoposaurus krasiejowensis*. Histological analyses of the skull of *Metoposaurus krasiejowensis*, additionally supported by Computational Modelling (CM) and Finite Element Analysis (FEA), granted new insights into dermal bone microstructure, origin and ecological implications among metoposaurids (Gruntmejer, Konietzko-Meier & Bodzioch, 2016 with Supplemental Material; Konietzko-Meier et al., 2018; Gruntmejer et al., 2019b). Gruntmejer, Konietzko-Meier & Bodzioch (2016) provided detailed characteristics of almost all cranial bones with preliminary data on their biomechanical resistance. Further studies have revealed that dermal bone microstructure is not only limited to the bone itself, but also relates to specific areas of the skull (Konietzko-Meier et al., 2018). Dominant within the skull roof, a thick and moderately porous bone with numerous, densely arranged Sharpey’s fibres shows that the skull was a robust structure which enabled the animal to bite actively on prey. Computational simulation of the skull of *Metoposaurus krasiejowensis* using FEA (Fortuny, Marcé-Nogué & Konietzko-Meier, 2017) and histological analysis of cranial sutures (Gruntmejer, 2012; Gruntmejer et al., 2019b) have documented that metoposaurids specialised in direct biting during ambush and active hunting. Moreover, previous studies which focused on skull biomechanics in *Metoposaurus krasiejowensis*, had already shown that histology and the computed Finite Elements (FE) method were not flawless research techniques. Histological analysis that focuses on the sections of single bones may provide a prediction of overall bone biomechanics (Gruntmejer, Konietzko-Meier & Bodzioch, 2016; Konietzko-Meier et al., 2018). Single bones may show various biomechanical resistance at its different portions. It has been confirmed in a skull of *Metoposaurus* that even the same bone may show various microstructures in different areas (Gruntmejer, Konietzko-Meier & Bodzioch, 2016). In a computational approach, FEA results are very often based on some simplifications and may thus mask significant biomechanical features, *i.e*., bone porosity or ornamentation (Bright, 2014; Konietzko-Meier et al., 2018; Gruntmejer et al., 2019b). However, applying both methodologies in parallel to a wider extent may provide a larger data set and more reliable results. Merged histological and computational FE studies have revealed that metoposaurids were well adapted to life in Late Triassic ecosystems and used two foraging techniques of prey capture, *i.e*., bilateral and lateral biting during active swimming and bilateral biting, while remaining buried in mud and attacking prey in ambush (Konietzko-Meier et al., 2018; Gruntmejer et al., 2019b).

In contrast to the skull, the mandible histology of *Metoposaurus* has up to date been studied only briefly (Gruntmejer, 2015). Further histological analyses of mandibular sutures have shown that the lower jaw was under the influence of a complex loading regime during feeding activities (Gruntmejer et al., 2019a). Differences in sutural morphology and numerous Sharpey’s fibres along the edges of adjacent bones suggest that the mandible was a strong and flexible structure (Gruntmejer et al., 2019a). To our knowledge, there are no comparable studies of the mandible of *Metoposaurus*, with the exception of osteological descriptions provided by Konietzko-Meier & Wawro (2007) and Sulej (2007). Additionally, the histology of mandibles in other early tetrapods is not yet known either.

Thus, the presentation of detailed histological characteristics of serially sectioned mandible bones may allow not only for a better understanding of metoposaurid palaeoecology in Late Triassic ecosystems, but also provide a solid basis for other research techniques. *e.g*., for FEA. Accumulations of skeletal elements of *Metoposaurus krasiejowensis* in the Upper Triassic bone-bearing bed at Krasiejów (southwest Poland) allow us to conduct histological analyses in a much more detailed way than is usually the case. Histology is an invasive research method; thus, it cannot be used when fossil material is rare or valuable. Generally, such studies are carried out on the basis of several thin sections at best. However, detailed investigations of serially sectioned pieces from a single individual have never been conducted previously for any vertebrate taxon.

The goals of the present study are threefold: (1) to document, for the first time, the mandible histology on the basis of serially sectioned specimens; (2) to compare the histological variability of mandibles of two conspecific individuals, and (3) to provide a preliminary interpretation of mandible biomechanics based on our histological framework.

## Materials and Methods

### Material

The material examined comes from an abandoned claypit near the village of Krasiejów in the Opole voivodship (Upper Silesia, southwest Poland). Geologically, this region is located along the south-easterly edge of the Fore-Sudetic Homocline and sedimentary strata at Krasiejów are of Late Triassic-Norian age, based on stratigraphical data (Racki & Szulc, 2014; Szulc, Racki & Jewuła, 2015a; Szulc et al., 2015b; Szulc, Racki & Bodzioch, 2017; Jewuła et al., 2019) or Carnian age, according to biochronological studies (Dzik & Sulej, 2007, 2016; Lucas, Spielmann & Hunt, 2007; Lucas, 2015).

**Figure 1:**
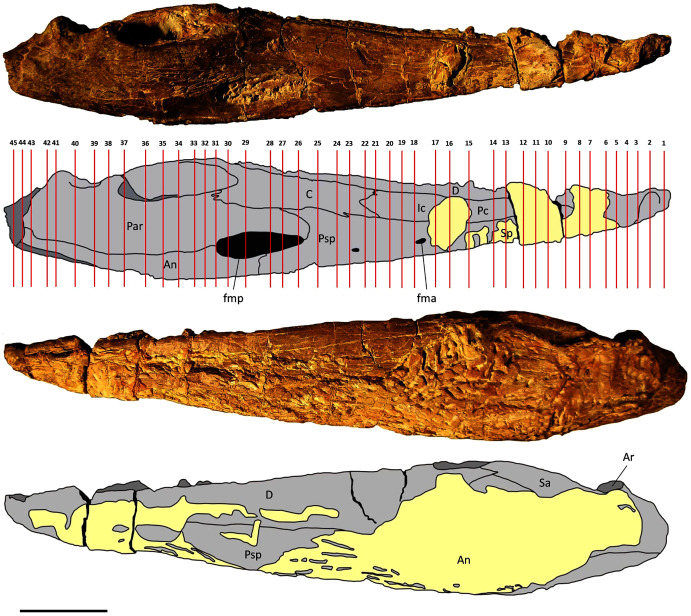
Mandible of *Metoposaurus krasiejowensis* in lingual and labial views (UOPB 01145) and schematic drawings with marked locations of sectioning planes (after Gruntmejer et al., 2019a). Grey colour indicates prepared parts of the mandible; yellow colour refers to matrix-covered areas. Scale bar equals 50 mm. Abbreviations: An, angular; Ar, articular; C, coronoid; D, dentary; Ic, intercoronoid; Par, prearticular; Pc, precoronoid; Psp, postsplenial; Sa, surangular; Sp, splenial; fma, anterior Meckelian foramen; fmp, posterior Meckelian foramen.

**Figure 2:**
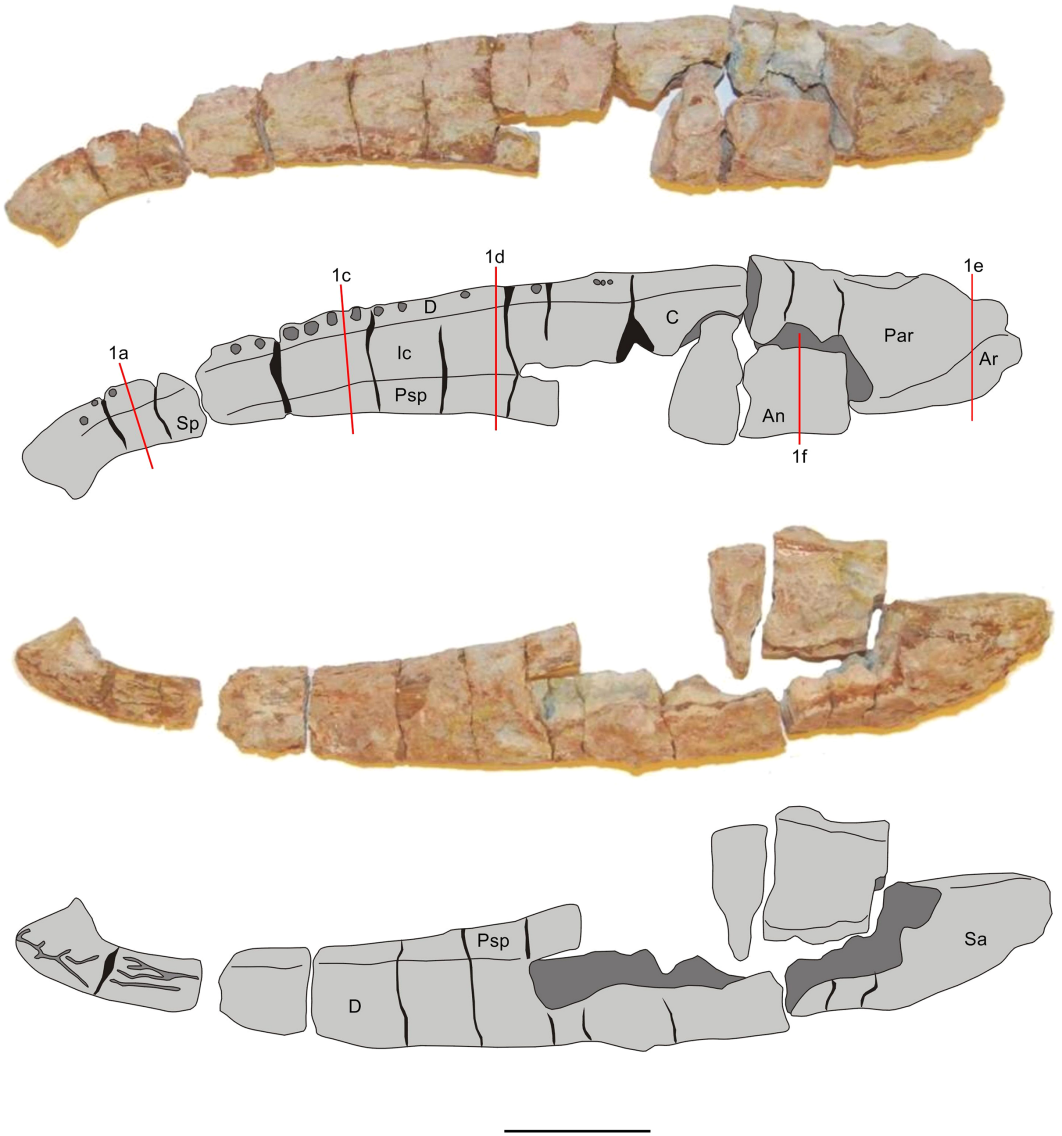
Mandible of *Metoposaurus krasiejowensis* in lingual and labial views (UOPB 01027) and schematic drawings with marked locations of sectioning planes (after Gruntmejer et al., 2019a). Grey colour indicates prepared parts of the mandible; yellow colour refers to matrix-covered areas. Scale bar equals 50 mm. Abbreviations: An, angular; Ar, articular; C, coronoid; D, dentary; Ic, intercoronoid; Par, prearticular; Psp, postsplenial; Sp, splenial.

Two mandibles of *Metoposaurus krasiejowensis* have been analysed histologically; both specimens are stored in the collections of the University of Opole, Institute of Biology, Laboratory of Palaeobiology (abbreviation: UOPB). UOPB 01145 is a complete (38 cm in length), well-preserved left ramus (Fig. 1), UOPB 01027 a near-complete (34 cm in length) right hemimandible (Fig. 2).

### Methods

In total, 50 transverse thin sections of the mandibles of *Metoposaurus krasiejowensis* have been studied. UOPB 01145 was sectioned in its entirety, at distances of less than 10 millimetres. In this way, 45 samples were prepared from a single specimen (Fig. 1). UOPB 01027 was sectioned in five specific areas of the mandible (*i.e*., symphyseal region, medial and postglenoid area) in order to compare the histological variability between the two specimens.

Thin sections were prepared at the Institute of Geology, Adam Mickiewicz University (Poznań, Poland). Following standard petrographical procedures (Lamm, 2013), thin sections were ground and polished to a thickness of 60–80 µm using wet *SiC* grinding powders (*SiC* 600, 800). Microscopic observations were conducted using a LEICA DMLP light microscope in plane and cross-polarised light and with a gypsum filter, and were supplemented with a scanning electron microscope (SEM) Hitachi S-3700N and binocular microscopes Olympus SZH10 and SZ61.

The histological nomenclature used in the present study follows Francillon-Vieillot et al. (1990), Witzmann (2009) and Lamm (2013). Histological characteristics of the mandible of *Metoposaurus* were assessed and compared to results of previous work on the cranial bone histology of this taxon (Gruntmejer, Konietzko-Meier & Bodzioch, 2016). Preliminary interpretations of biomechanical functions for bones that were examined histologically are those of Konietzko-Meier et al. (2018). In view of the large number of thin sections and high histovariability along the mandible of *Metoposaurus krasiejowensis*, it would be difficult to describe the histology of each bone individually. Instead, their histology and microstructure are briefly presented in Table 1. Thus, in the present study, we provide a description of the histological variability along the entire mandible arch.

**Table 1:** General histology of *Metoposaurus krasiejowensis* mandible of UOPB 01145 and UOPB 01027 specimens.

Bone	Bone thickness	Ornamentation	Growth marks	Sharpey’s fibers	Vascularization	Bone remodelling
Angular (thin sections no. 22–45) UOPB 01145	2–10 mm	high ridges (5 mm)	alternation of vascularised zones and avascular annuli	very numerous; two types: (1) long and thick, (2) thin and short	low (almost avascular in the middle part of the bone) to very high (numerous secondary osteons in posterior part of the bone)	low (numerous erosion cavities but in small sizes) to very high (numerous and large erosion cavities)
Angular (thin sections no. 1e and 1f) UOPB 01027	3–10 mm	high ridges (5 mm)	visible thick zones and thinner annuli	very numerous; two types: (1) long and thick, (2) thin and short	low (almost avascular in the middle part of the bone) to very high (numerous secondary osteons in posterior part of the bone)	low (numerous erosion cavities but in small sizes) to very high (numerous and large erosion cavities)
Articular (thin section no. 41–44) UOPB 01145	over 10 mm at the widest place	lack	not visible	not visible	low (simple vascular canals and secondary osteons)	highly porous trabecular region
Articular (thin section no. 1e) UOPB 01027	around 10 mm	lack	not visible	not visible	low (simple vascular canals and secondary osteons)	highly porous trabecular region
Coronoid (thin sections no. 22–31) UOPB 01145	2–8 mm	lack	not visible	very short and rare	moderate (several secondary osteons)	low (presence of few and small erosion cavities)
Coronoid (thin section no. 1d) UOPB 01027	1–3 mm	lack	not visible	very short and rare	moderate (several secondary osteons)	low (presence of few and small erosion cavities)
Dentary (thin sections no. 1–29) UOPB 01145	2–10 mm	generally lack; only three ridges at symphyseal region	alternation of vascularised zones and avascular annuli	very numerous; two types: (1) long and thick, (2) thin and short	high (numerous secondary osteons and vascular canals)	moderate (numerous erosion cavities but in small sizes)
Dentary (thin sections no. 1a, 1c and 1d) UOPB 01027	3–6 mm	generally lack; only two ridges at symphyseal region	visible thick zones and thinner annuli	very numerous; two types: (1) long and thick, (2) thin and short	high (numerous secondary osteons and vascular canals)	moderate (numerous erosion cavities but in small sizes)
Intercoronoid (thin sections no. 15–21) UOPB 01145	3 mm	lack	not visible	very short and rare	moderate (several secondary osteons)	low (presence of few and small erosion cavities)
Intercoronoid (thin section no. 1c) UOPB 01027	3 mm	lack	not visible	very short and rare	moderate (several secondary osteons)	low (presence of few and small erosion cavities)
Prearticular (thin sections no. 26–44) UOPB 01145	1–10 mm	lack	not visible	short and numerous	low to moderate (several secondary osteons)	moderate (numerous erosion cavities but in small sizes) to very high (numerous and large erosion cavities)
Prearticular (thin sections no. 1e and 1f) UOPB 01027	4–8 mm	lack	not visible	not visible	low to moderate (several secondary osteons)	moderate (numerous erosion cavities but in small sizes) to very high (numerous and large erosion cavities)
Precoronoid (thin sections no. 8–14) UOPB 01145	4 mm	lack	not visible	very short and rare	moderate (several secondary osteons)	low (presence of few and small erosion cavities)
Precoronoid (thin section no. 1a) UOPB 01027	3 mm	lack	not visible	very short and rare	moderate (several secondary osteons)	low (presence of few and small erosion cavities)
Postsplenial (thin sections no. 16–28) UOPB 01145	2–8 mm	medium high ridges (3 mm)	alternation of vascularised zones and avascular annuli	very numerous; two types: (1) long and thick, (2) thin and short	moderate to high (numerous secondary osteons and vascular canals)	moderate (numerous erosion cavities but in small sizes)
Postsplenial (thin sections no. 1c and 1d) UOPB 01027	3–6 mm	medium high ridges (2 mm)	alternation of vascularised zones and avascular annuli	very numerous; two types: (1) long and thick, (2) thin and short	moderate to high (numerous secondary osteons and vascular canals)	moderate (numerous erosion cavities but in small sizes)
Splenial (thin sections no. 4–16) UOPB 01145	2–7 mm	moderate high ridges (3 mm)	not visible	visible but not numerous; thin and short	low to moderate (simple vascular canals and secondary osteons)	low to moderate (simple erosion cavities)
Splenial (thin section no. 1a) UOPB 01027	4 mm	lack	not visible	visible but not numerous; thin and short	low (simple vascular canals and secondary osteons)	low (lack of erosion cavities)
Surangular (thin sections no. 31–44) UOPB 01145	2–10 mm	generally lack; simple medium high ridges (3 mm)	alternation of vascularised zones and avascular annuli	very numerous; two types: (1) long and thick, (2) thin and short	low (almost avascular in the middle part of the bone) to very high (numerous secondary osteons in upper part of the bone)	low (presence of few and small erosion cavities) to very high (numerous and large erosion cavities)

A number of other problematic issues need to be addressed in order to understand correctly the histological characterisation of some bone components, *i.e*., external and internal cortices. The mandible of *Metoposaurus* is a tube-like structure, in which several bones (dentary, splenial, postsplenial and angular) possess variable shapes along their length. In many places they become U-shaped in cross section; thus, recognition of the position of external and internal cortices may be difficult. In the present study, it is assumed that term ‘external cortex’ refers to parallel-fibred bone which occurs in the uppermost and ornamented parts of the bones on the labial side and similarly in the uppermost and unornamented parts of the same bones on the lingual side. Conversely, ‘internal cortex’ refers to parallel-fibred bone which comprises the opposite and unornamented margins of bones, both on the labial and lingual side. To avoid confusion, we use the term ‘growth marks’ in relation to lines of arrested growth (LAGs), resting lines, zones and annuli (Francillon-Vieillot et al., 1990). A line of arrested growth (LAG) usually is interpreted as an annual episode of a growth cessation linked, for instance, with unfavorable external conditions (Francillon-Vieillot et al., 1990). Resting lines are also expressions of growth stops, that could occur several times during a year. This is a well-known phenomenon in long bones of *Metoposaurus*, in which numerous resting lines could be observed in a single annulus (Konietzko-Meier & Sander, 2013). Alternations of thick zones and thinner annuli illustrate periods of faster or slower growth. Moreover, two types of collagen fibres are described in the present work: Sharpey’s fibres, *i.e*., structures which indicate the point of soft tissue attachment to the bone (muscles, tendons or skin), and interwoven structural fibres (ISF), which indicate the metaplastic origin of dermal bones (Scheyer & Sander, 2004; Scheyer & Sánchez-Villagra, 2007).

For the most representative cross sections used herein (*i.e*., numbers 4, 14, 22, 29, 36, 39, 41 and 43), bone porosity was calculated using the pixel-counting software ‘bw-counter’ developed by Peter Göddertz at the Institute of Geoscience of the University of Bonn (© Peter Göddertz, IGPB). All cross sections are considered to represent a single sample record, without distinction of individual bones. Only the articular, visible in the two final sections, was invariably calculated separately because of the different origin and structure.

## Results

The mandible of *Metoposaurus* is longitudinally tubular in shape and structure, consisting of a set of 10 bones that vary in size and shape (Figs. 1 and 2). With the exception of the articular, all mandibular bones are dermal in origin in that they ossify directly within the dermis without a cartilaginous predecessor. Their external surface is ornamented by grooves and longitudinal ridges, similar to those of the skull bones and pectoral girdle. Dermal bones show a diploë structure in cross section with a well-differentiated compact external and internal cortex which are separated by a wide and porous middle region. Bone thickness varies from around two to over 10 millimetres along the mandible, even within the same bones, such as the angular or surangular (Table 1). The exception is the articular bone, which undergoes an endochondral ossification process.

### Histology of dermal bones

**Figure 3:**
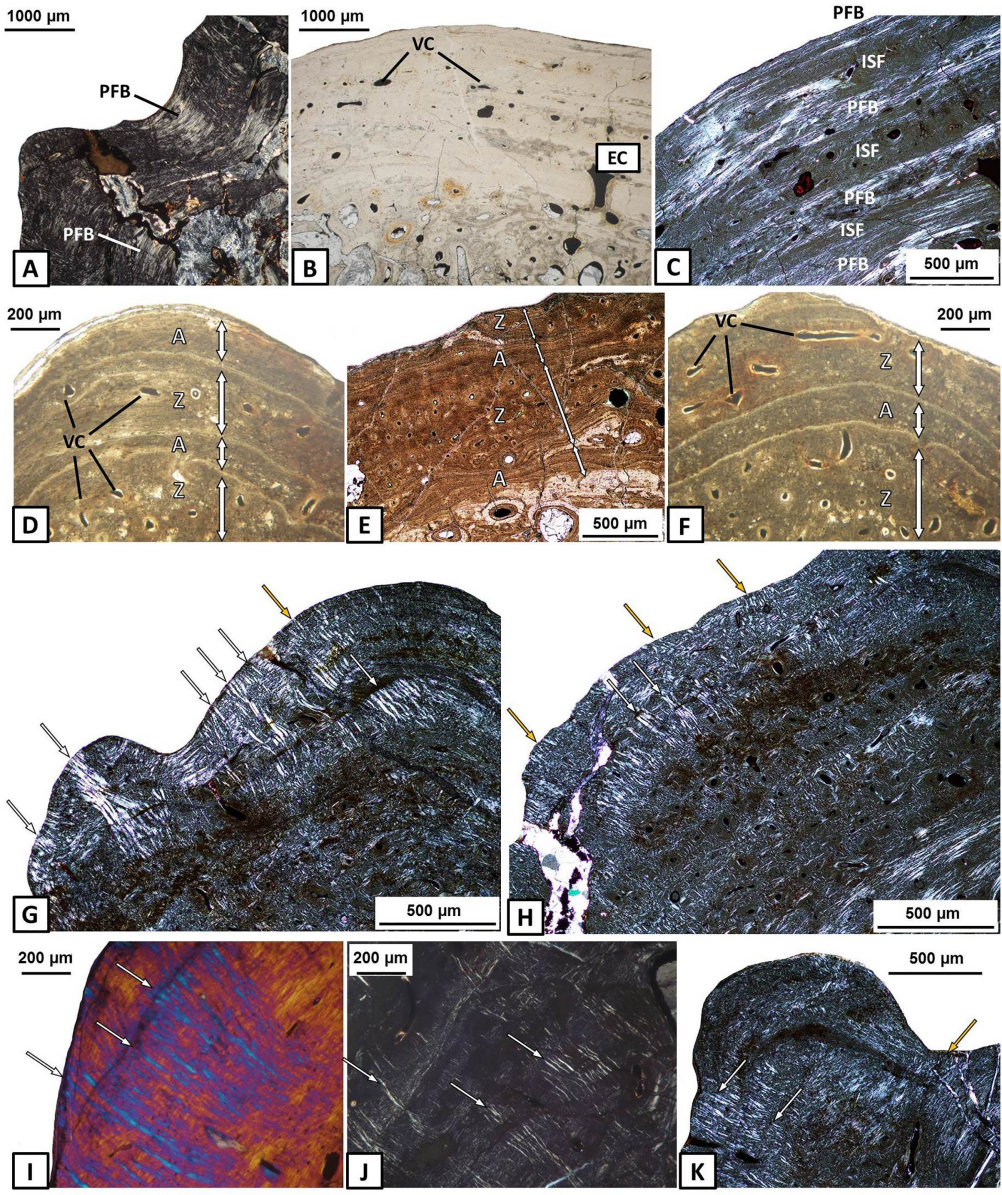
Histology of the external cortex of the mandible bones of *Metoposaurus krasiejowensis* (UOPB 01145 and UOPB 01027). (A) Thick layer of parallel-fibred bone in ornamented part of the dentary; (B) Compact external cortex of the splenial with several vascular canals (VC); (C) Alternation of parallel-fibred bone (PFB) and interwoven structural fibres (ISF) of the splenial; (D–F) Growth marks structure (indicated by arrows) visible as thick and vascular zones, separated by avascular annuli along the dentary; (G–H) Two types of well-mineralised Sharpey’s fibres of the dentary, which indicate the skeletal muscle attachment (white arrows) and the periosteum connection (yellow arrows); (I) Long Sharpey’s fibres (white arrows); (J–K) Sharpey’s fibres (white arrows) in subsurface and deeper part of the external cortex of the angular. Images (I) in gypsum filter, (B) and (D–F) in plane polarised light, others in cross-polarised light. Abbreviations: A, annulus; EC, external cortex; ISF, interwoven structural fibres; PFB, parallel-fibred bone; VC, vascular canals; Z, zone.

The external cortex consists usually of compact parallel-fibred bone with a thickness of 2 millimetres (Figs. 3A–3B). In some areas, Interwoven Structural Fibres (ISF) are visible in the external cortex (Fig. 3C). Growth marks appear as thick and well-vascularised zones, separated by thinner and avascular annuli (Figs. 3D–3F), in both the sculptural ridges (labial side) and unornamented parts of the external cortex (lingual side), especially in the dentary and the angular. Typical lines of arrested growth (LAG) do not occur. Sharpey’s fibres are numerous, well-mineralised and densely packed along the entire length of the mandible and are present in all dermal bones. However, they are the most common on the labial side of the mandible, mainly in the ornamented parts of the dentary, angular and surangular (Figs. 3G–3H). In these areas Sharpey’s fibres are very long and penetrate into the deeper parts of the external cortex (Figs. 3I–3K). In bones on the lingual side of the mandible, especially in the precoronoid, intercoronoid, coronoid and postsplenial, Sharpey’s fibres are shorter and thinner.

**Figure 4:**
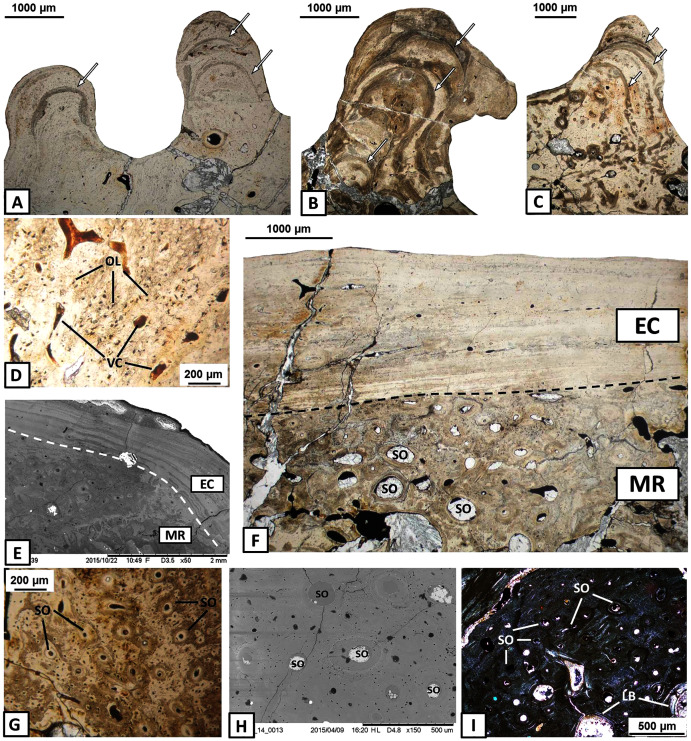
Histology of the external cortex and middle region of the mandible bones of *Metoposaurus krasiejowensis* (UOPB 01145 and UOPB 01027). (A) Alternation of growth rate pattern (indicated by arrows) within sculptural ridges of the splenial; (B) the same, postsplenial; (C) the same, angular; (D) Vascular canals and numerous osteocyte lacunae in the deeper portion of the external cortex; (E) Sharp transition from external cortex into the middle region of the dentary (indicated by dashed line); (F) Same as in E, postsplenial; (G–I) Dense clusters of small secondary osteons in the upper areas of the middle region. Images (E) and (H) are SEM images, (I) in cross-polarised light, others in plane-polarised light. Abbreviations: EC, external cortex; LB, lamellar bone; MR, middle region; OL, osteocyte lacunae; SO, secondary osteons; VC, vascular canals.

The external cortex is poorly vascularised. A high number of vascular canals and small primary osteons occur only in thick zones, mainly in the dentary and splenial. On the labial side, sculptural ridges are almost avascular in the postsplenial and angular, and alternations with an earlier generation of ridges are present (Figs. 4A–4C). Osteocyte lacunae with branched canaliculi occur numerously along the external cortex in all bones examined (Fig. 4D).

**Figure 5:**
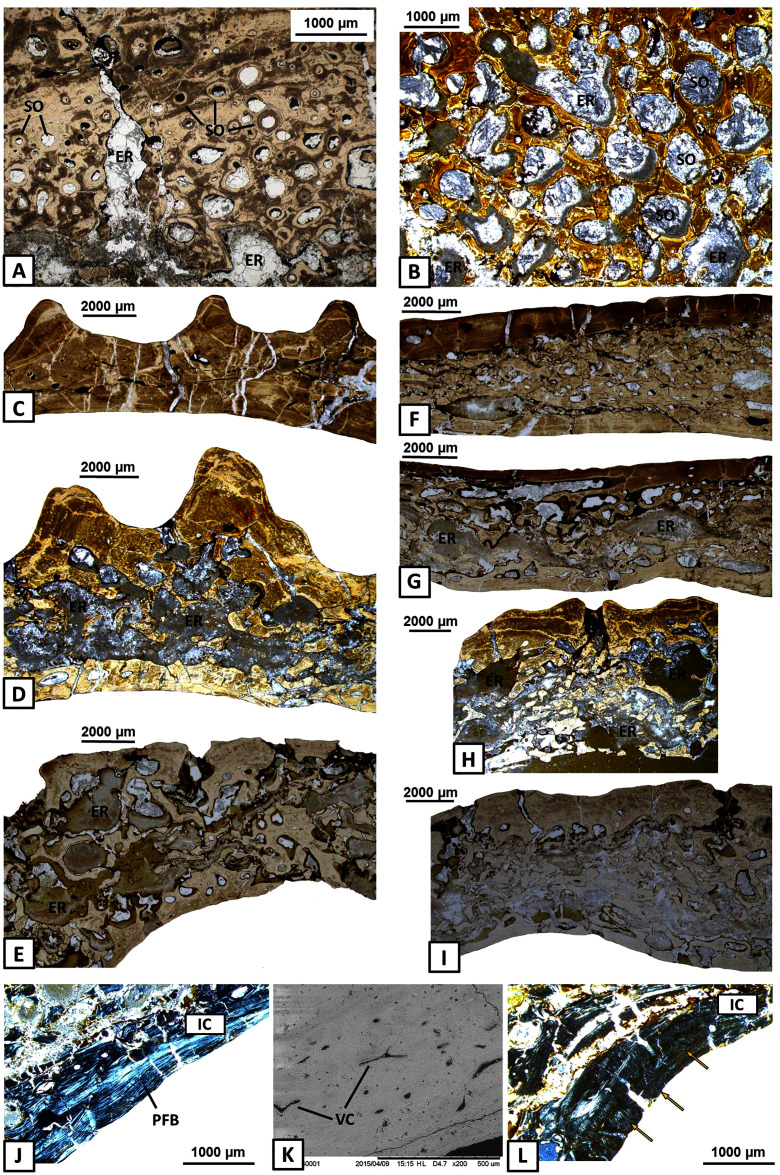
Histology of the middle region and internal cortex of the mandible bones of *Metoposaurus krasiejowensis* (UOPB 01145 and UOPB 01027). (A) Highly remodelled and well-vascularised parts of the surangular; (B) Dense clusters of large secondary osteons of the angular; (C–E) Microstructural variability along the angular; (F–G) Microstructural variability along the prearticular; (H–I) Microstructural variability at the sutural contact between angular and surangular; (J) Internal cortex with well-differentiated, parallel-fibred bone of the angular; (K) Longitudinal vascular canals within internal cortex; (L) Dense clusters of short Sharpey’s fibres (yellow arrows) along the internal cortex of the angular. Image (K) is an SEM image, (J) and (L) in cross-polarised light, others in plane polarised light. Abbreviations: ER, erosion cavities; IC, internal cortex; PFB, parallel-fibred bone; SO, secondary osteons; VC, vascular canals.

The external cortex clearly transits into the extensive middle region which covers the larger parts of every dermal bone in cross section (Figs. 4E–4F). The vascular network in the middle region varies from poorly developed to well developed. Small secondary osteons and primary vascular canals occur numerously next to the border between the middle region and the external cortex (Figs. 4G–4I). In many areas, dense clusters of small osteons create the beginning of Haversian tissue. These features are clearly seen, mainly in the posterior part of the dentary (Fig. 4G), the anterior areas of the angular and occasionally in the splenial and the postsplenial (Figs. 4H–4I). The deeper parts of the middle region are occupied by larger secondary osteons (50–200 µm in diameter) which create dense clusters mainly in the posterior parts of the postsplenial, angular and surangular (Figs. 5A–5B). The middle region is highly remodelled. Very large and irregularly shaped erosion cavities occur here. The degree of porosity may drastically change even along a single bone. For instance, in the angular, the anterior part is relatively thin and of low porosity, whereas in its distal parts it becomes thicker and also highly porous (Figs. 5C–5E). A similar pattern is present in the prearticular (Figs. 5F–5G) and surangular (Figs. 5H–5I). Posteriorly, the middle part of these bones is almost absent, and erosion cavities could exceed 3 millimetres in length.

The internal cortex consists of parallel-fibred bone and its thickness usually reaches around one millimetre (Fig. 5J). Vascularisation is moderate: longitudinal primary vascular canals are very numerous (Fig. 5K). Small secondary osteons are less common and filled with a thin layer of lamellar bone. Sharpey’s fibres are long, dense and packed in bundles; they occur along the subsurface and in the deeper part of the internal cortex, especially in the dentary and angular (Fig. 5L). Osteocyte lacunae are numerous as well. Growth marks are not visible.

### Histology of the articular

**Figure 6:**
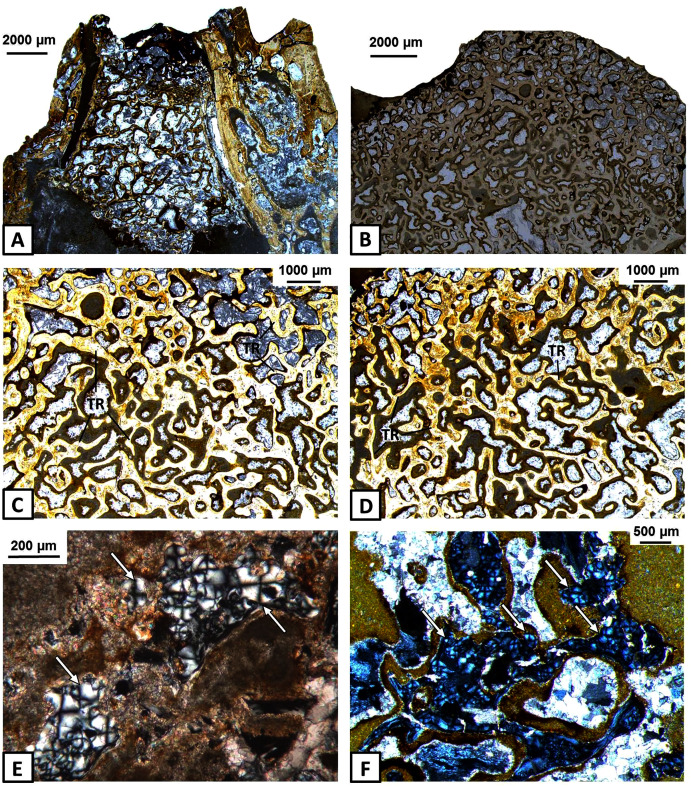
Histology of the articular of *Metoposaurus krasiejowensis* (UOPB 01145 and UOPB 01027). (A) General microstructure of UOPB 01027 specimen; (B) and of UOPB 01145 specimen; (C–D) Bone trabeculae in the central part of the articular; (E–F) Numerous remains of calcified cartilage visible in the trabeculae (arrowed). Images (E) and (F) in cross-polarised light, others in plane polarised light. Abbreviations: TR, bone trabeculae.

The articular consists of extensive spongy bone, which is surrounded by a thin cortex (Figs. 6A–6B). Poorly preserved and avascular cortex consists of parallel-fibred bone. Growth marks and Sharpey’s fibres are not visible. The middle region consists of extensive spongy bone with large cavities between trabeculae (Figs. 6C–6D). Within the bony trabeculae, numerous and elongated osteocyte lacunae are present and remains of calcified cartilage are visible (Figs. 6E–6F).

### Histological variability of the mandible

**Figure 7:**
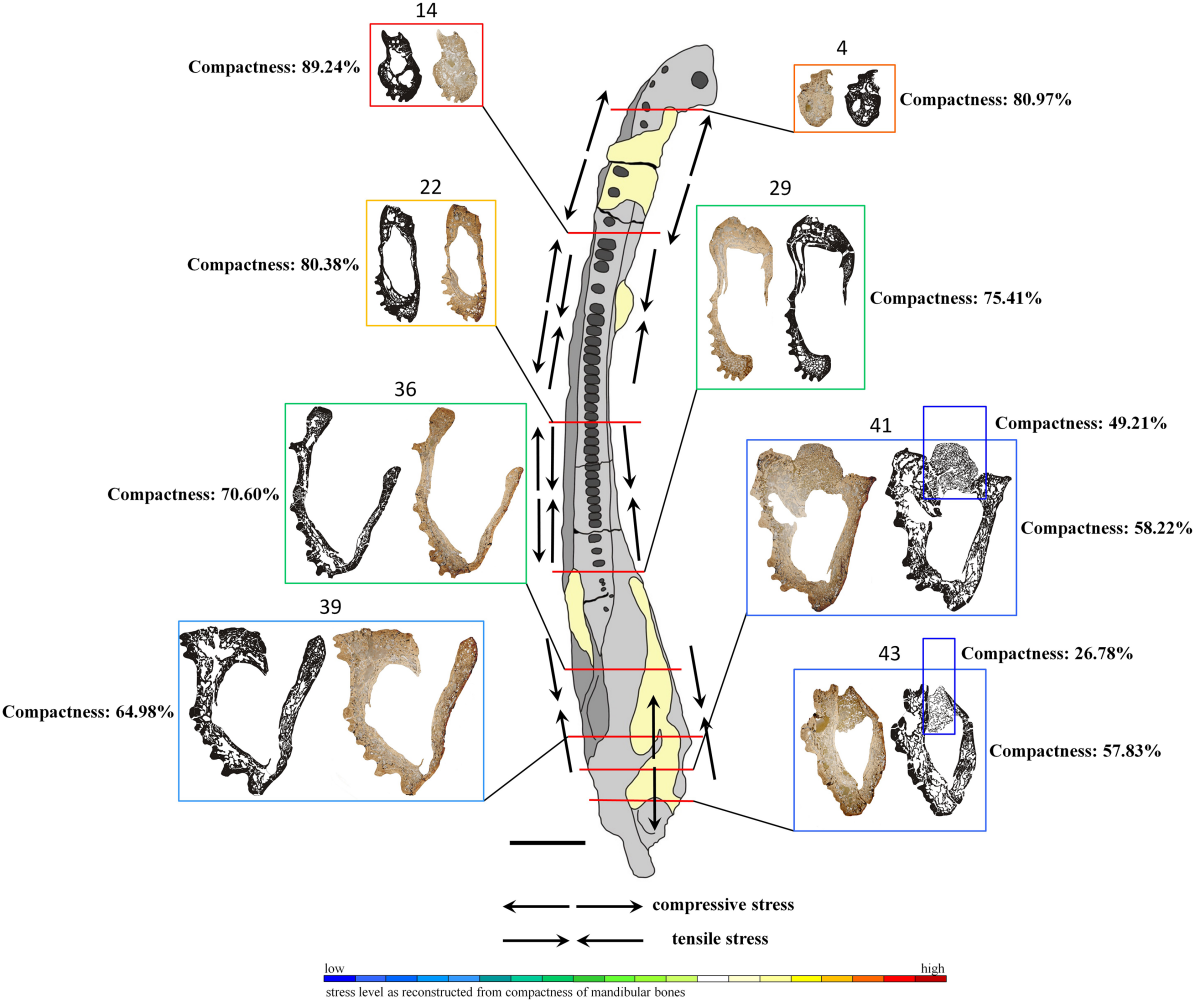
General microanatomy of selected thin sections of specimen UOPB 01145 and estimated biomechanical loading reconstructed on the basis of the microstructural characteristics of the *Metoposaurus krasiejowensis* mandible in top view. Black and white images of selected thin sections show calculated compositions of bone thickness and its compactness. Colour frames and scale bar represent estimated values of stress distribution in different parts of the mandible, but they only suggest a contractual stress regime (higher or lower). Black arrows indicate places underwent various stresses (tensile-compressive) caused by mandible suture mechanics, followed by Gruntmejer et al. (2019a). Black scale bar equals 40 mm.

In the lower jaw of *Metoposaurus krasiejowensis*, the bone microanatomy and histological framework are highly variable. Symphyseal and anterior parts of the mandible represent a compact bony structure (calculated porosity below 20%—Table 1; Fig. 7) being a conglomerate of bones of low porosity, *i.e*., in the dentary, splenial, precoronoid and the anterior part of intercoronoid and postsplenial. On the labial side, the dentary, precoronoid and splenial possess a well-developed vascular network (Figs. 3D–3F and 4D) and abundant clusters of Sharpey’s fibres (Figs. 3G–3K). In the median part, the mandible is formed as a tube-like structure with a large Meckelian canal (sectioning sample no. 17–32 of UOPB 01145). Bone thickness varies across this part of the mandible. Dentary thickness varies between 1 and 5 millimetres, as in the coronoid, whereas in the postsplenial and angular it varies between 2 millimetres and almost 10 millimetres (Table 1). The porosity of these bones is low to moderate (between 20% and 30%, Table 1; Fig. 7). However, the most widely variable factor is the degree of vascularisation. Dense clusters of large, secondary osteons are visible in the thickest parts of the postsplenial and angular (Figs. 5A–5B). Distinct variations in bone thickness and compactness start in the area of the glenoid foramen (sectioning sample no. 33–45 of UOPB 01145). The average thickness of the surangular and angular is around 4 millimetres. However, in the upper part of the surangular and the posterior area of the angular, the thickness of these bones can reach even up to 10 millimetres. The prearticular thickness varies from 2 to 8 millimetres. The porous nature of all of these bones becomes very marked in contrast to the anterior and median areas of the mandible. The extensively remodelled middle region consists of large and irregular erosion cavities which can reach around 3 millimetres in length. The microanatomical variability could be easily followed, for instance in the angular (Figs. 5C–5E), prearticular (Figs. 5F–5G) and surangular (Figs. 5H–5I). These bones are thinner but more compacted in their anterior parts, while posteriorly they become much thicker and more porous. The high porosity makes documentation of histological characteristics difficult in the glenoid and postglenoid area.

### Microstructure of the mandible bones

The overall thickness of bone walls increases along the mandible from the symphyseal to postglenoid area (Table 1). The symphyseal part is a tube-like structure, relatively massively built with only a few, small cavities. The calculated porosity in this area equals about 19% (Fig. 7). The highest compactness is observed in slide 14, where it reaches (89%). Posteriorly, the diameter of the entire mandible ramus increases rapidly, with a size increase of the inner cavities and a decrease of bone thickness. Simultaneously, bone porosity growths, attaining 42% in slide 43 (Fig. 7). The articular shows a different structure, with a very loose framework, the bone porosity reaching up to 51% in slide 41 and over 73% in slide 43.

## Discussion

### Skeletochronology

In contrast to long bones in which growth mark structure could be easily determined and followed (Konietzko-Meier & Klein, 2013; Konietzko-Meier & Sander, 2013; Teschner, Sander & Konietzko-Meier, 2018; Teschner et al., 2020), dermal bones are not a good source of skeletochronological data (Gruntmejer, Konietzko-Meier & Bodzioch, 2016). These structures appear in the skull of *Metoposaurus* only as resting lines and alternations of thick zones and annuli. Typical lines of arrested growth (LAGs) could not be found (Gruntmejer, Konietzko-Meier & Bodzioch, 2016). Similar skeletochronological patterns occur along the mandibles of *Metoposaurus*. Lines of arrested growth are not visible. However, very common is the alternation of thick zones, separated by thinner and avascular annuli (Figs. 3D–3F). The presence of this alternation is not constant, but yet can clearly be followed along the dentary and angular. Highly vascularised zones are associated with more rapid growth of an organism which can be linked to favourable environmental conditions with high water levels and increased food availability (Konietzko-Meier & Sander, 2013). Avascular annuli are related to less beneficial seasons when life activity and body growth were slower (Konietzko-Meier & Sander, 2013). Due to the lack of continuity of growth marks along the mandible bones and high bone remodelling, it is difficult to assess the individual age of specimens studied; the same holds true for the skull (Gruntmejer, Konietzko-Meier & Bodzioch, 2016).

### Ossification processes of the mandible

Dermal bones develop directly from the soft tissue, *i.e*., the deep parts of the dermis without periost, osteoblasts and osteoid, *via* metaplastic ossification (Francillon-Vieillot et al., 1990; Vickaryous & Hall, 2008). In temnospondyls, dermal bones form the skull, mandible and pectoral girdle (two clavicles and an interclavicle), the external surfaces of which are ornamented by grooves, ridges and polygonal pits (*e.g*., Von Meyer, 1858; Fraas, 1889; Fritsch, 1889; Zittel, 1911; Witzmann, 2009; Witzmann et al., 2010; de Buffrénil et al., 2016). In cross section, the dermal bone shows a diploë structure. Their external surface creates a compact layer of an intramembranous component which appears as parallel-fibred bone, lamellar bone or interwoven structural fibres (Scheyer & Sander, 2004; Scheyer & Sánchez-Villagra, 2007). The external cortex gradually passes into an extensive and highly porous middle region, usually with numerous clusters of secondary osteons (Witzmann, 2009). The internal cortex also consists of an intramembranous, lower part of the bone. In mandibles of *Metoposaurus* (specimens UOPB 01145 and UOPB 01027), dermal bones also represent a diploë combination in cross section. External and internal cortices usually consist of a compact layer of parallel-fibred bone (Figs. 3A–3C and 5J). Its metaplastic origin additionally confirms the presence of interwoven structural fibres (ISF) in the subsurface part of the cortex and along sutural margins of the surangular and prearticular (Gruntmejer et al., 2019a). The middle region is highly remodelled and forms the largest portion of the bone (Figs. 5C–5I).

Endochondral bones ossify throughout a cartilaginous predecessor and consist of a trabecular region, surrounded usually by a thin layer of cortex. In the skull of *Metoposaurus*, the quadrate and exoccipital are endochondral bones (Gruntmejer, Konietzko-Meier & Bodzioch, 2016). In the case of the mandible, the articular does not constitute a dermal bone in origin, this having formed of a thin layer of avascular cortex and a very extensive, trabecular region (Figs. 6A–6D). Remnants of calcified cartilage show an endochondral development of articular bone (Figs. 6E–6F).

### Sharpey’s fibres

Dense clusters of two types of Sharpey’s fibres have been recognised in long bones of *Metoposaurus* (Konietzko-Meier & Sander, 2013). Thick and long fibres are indicative of points of attachment of strong skeletal muscles and tendons, whereas shorter ones reflect areas in which the periosteum is connected to the bone. A similar distribution of Sharpey’s fibres can be observed within the skull of *Metoposaurus* (Gruntmejer, Konietzko-Meier & Bodzioch, 2016). Short fibres are rare, but they do appear along the length of the skull and may indicate a delicate, soft-tissue attachment. More numerous and thick Sharpey’s fibres occur in the posterior part of the skull in the tabular and occipital condyle. The presence of well-mineralised and dense bundles in these areas suggests a strong skeletal muscle attachment of the skull to the vertebral column (Gruntmejer, Konietzko-Meier & Bodzioch, 2016). Moreover, numerous clusters of short Sharpey’s fibres occur at sutural edges between adjacent bones (Gruntmejer et al., 2019b). Their presence along the lateral edges of dermal bones refers to the occurrence of collagen fibres within the sutural morphospace which bridged adjacent bones and increased the connections between them (Rafferty & Herring, 1999).

In mandibles of *Metoposaurus* (specimens UOPB 01145 and UOPB 01027), both types of Sharpey’s fibres are present as well. However, long and short fibres occur together along the whole length of the mandible on the labial side (Figs. 3G–3K). Their presence was noted in sculptural ridges and the unornamented parts of bones where longer Sharpey’s fibres penetrate into the deeper parts of the external cortex. Thinner fibres are present along the lingual side of the mandible and in the internal cortex of several bones (Fig. 5L). Dense clusters of two types of Sharpey’s fibres along the labial part of the mandible suggests abundant attachment of skeletal muscles, tendons and ligaments on this side of the lower jaw. In contrast, the occurrence of shorter fibres along the lingual part of the mandible may relate to delicate and soft-tissue attachment which filled the inner surface of the mouth. Similar to the case of the skull, short and numerous Sharpey’s fibres were also noted along the sutural edges between adjacent bones of the mandible (Gruntmejer et al., 2019a). Moreover, their orientation is an important indicator for deduction of stress distribution during feeding activity (Rafferty & Herring, 1999; Herring & Teng, 2000; Jasinoski et al., 2010). Sutural morphology and crucial orientation of Sharpey’s fibres in the anterior part of the dentary and at the sutural contacts between the articular, surangular and prearticular suggest tensile stress in these areas. The tensile loading regime in the symphyseal and postglenoid region could be an adaptation to open the jaw very wide during predation (Gruntmejer et al., 2019a).

### Biomechanical condition of dermal bones in the mandible

Histological studies of dermal skull bones in *Metoposaurus krasiejowensis* have revealed that parameters such as bone thickness and porosity are the most variable along the skull and play an important role in its biomechanics (Konietzko-Meier et al., 2018). Bone thickness and compactness are genuine responses to stress distribution, *i.e*., bones lose strength and stiffness with increasing porosity; however, this can be partly compensated by an increase of structural thickness (Witzmann, 2009; Rinehart & Lucas, 2013). In addition to this, the histological properties are not related only to the bone, but strictly depend on the particular plane of sectioning (Gruntmejer, Konietzko-Meier & Bodzioch, 2016; Konietzko-Meier et al., 2018). In the skull of *Metoposaurus krasiejowensis* bones along the sagittal axis, such as the jugal and postorbital, sectioned along the sutural contact, represent the same histological framework and very good biomechanical properties (high thickness and low porosity) for stress resistance. Another example is the squamosal, sectioned in its anterior part and next to the otic notch, which showed different histological levels. Bones situated on the palatal side (*e.g*., vomer and pterygoid) display weaker biomechanical resistance, characterised by a low bone thickness and extreme porosity (Gruntmejer, Konietzko-Meier & Bodzioch, 2016). This suggests that the histological framework is not specifically limited to bone, but does reveal specific areas of the skull which probably were involved during feeding (Konietzko-Meier et al., 2018).

The wide range in microstructure of the mandible bones of *Metoposaurus krasiejowensis* may also provide preliminary data on its biomechanics. Similar to the skull, the microstructure of the mandible is not limited to a single bone either, but refers to a specific area of the mandible. The angular, sectioned in different areas along its length, shows a widely varying histological framework, from low thickness and high compactness to great thickness and an extremely high degree of porosity (Figs. 5C–5E). A similar microstructural pattern could be observed in the prearticular (Figs. 5F–5G). Moreover, some bones, such as the angular and surangular, sectioned along the sutural contact, represent the same histological framework (*i.e*., degree of bone thickness and compactness) which varies in other parts of these bones (Figs. 5H–5I).

Based on such high histovariability along the mandible of *Metoposaurus*, a preliminary interpretation of its biomechanics can be put forward. The symphyseal and other anterior parts of the mandible are characterised by low bone porosity which creates a thick conglomerate without any large cavity (Meckelian canal) between them (Fig. 7). Such microstructure may represent good biomechanical resistance of the anteriormost area of the mandible. A stable histological framework, accompanied by sutures that are related mostly to tensile loading (Gruntmejer et al., 2019a), suggests that the symphyseal part of the mandible was well adapted to resist stress created during feeding. The most stable structure is seen in slide 14, with the lowest porosity of the dentary, splenial and postsplenial and a complex structure without large inner cavity. This makes this part of the mandible a key point with the highest loading. In midline areas, bone thickness is low, reaching around 2–5 millimetres, but it increases to even 1 cm in sculptured regions. However, the compactness of these bones is lower than in the anterior part, thus biomechanical resistance of the central parts of the mandible can be assessed as moderate. A distinct increase of bone porosity begins in the glenoid area, whereas in postglenoid parts, all bones become extremely porous (up to 42% in slide 43) at thicknesses varying between 5 millimetres and 10 millimetres. However, the high level of porosity suggests the biomechanical parameters of these bones to have been moderate to low. Based on such varying microstructure among dermal bones, the mandible in general appears to be not such a strong structure. However, seemingly weak bones (caused by low compactness) could be strengthened by connections of strong skeletal muscles and other soft tissues. Dense clusters of thick and long Sharpey’s fibres support this assumption. Moreover, five types of sutures adapted to resist different stresses (tension *vs*. compression) have been noted along the mandible (Gruntmejer et al., 2019a). One of the biomechanical roles of sutures is to minimalise loading regimes acting within the skull or on the mandible during feeding. Thus, the complexity of sutural morphology and the abundance of Sharpey’s fibres along the mandible may compensate for the relatively weak microstructure of dermal bones of the mandible. These preliminary interpretations of mandible biomechanics should be worked out further and supplemented by additional studies, *e.g*., by computational modelling using finite element analysis. Based on previous works which focused on comparisons between FEA and histological results (Konietzko-Meier et al., 2018; Gruntmejer et al., 2019b), we know neither of these methods is flawless. For obtaining the most reliable results it is necessary to conduct investigations using histology as well as FEA in parallel.

## Conclusions

Dermal bones have already been studied histologically in temnospondyls, but not yet in serially sectioned specimens. The histology of cranial bones and bones of the pectoral girdle in metoposaurids has been described by several previous authors, but our current knowledge of mandible microstructure is poor. In the present study, a detailed histological description of the mandible of *Metoposaurus krasiejowensis* is presented. All bones represent a metaplastic origin with a uniform diploë structure, typical of dermal bones, with the exception of the articular. The external cortex consists of relatively thick parallel-fibred bone with well-differentiated growth mark structures and long Sharpey’s fibres. The extensive middle region represents a high level of bone remodelling with numerous secondary osteons and larger erosional cavities. The internal cortex consists almost solely of avascular parallel-fibred bone. The articular represents an endochondral development with a trabecular region and numerous cartilage remains. The same histological framework in specific areas of the mandible was confirmed in the two specimens compared herein.

The general histology of the mandible of *Metoposaurus krasiejowensis* represents a microanatomy that is similar to the one recognised in the skull, but with some differences. Dermal bones show a cyclical repetition of thick and well-vascularised zones that are separated by thinner and avascular annuli. However, this alternation is not uniformly consistent along the mandible and is therefore not a good source of skeletochronological data. Sharpey’s fibres are very long and occur in dense bundles along the whole mandible, both on the labial and lingual side. Such an abundance of clustered fibres suggests strong muscles and other soft-tissue attachments. The highly variable porosity of all bones, starting from the symphyseal to the articular region, also confirms previous results that bone microstructure is associated to specific areas of the mandible as an answer to local biomechanical conditions. The anterior part of the mandible is characterised by low bone porosity, thus this area experienced heavy loading during prey capture in contrast to the highly porous bones in the posterior and articular regions. The histology of mandibular sutures supports this assumption. Sutures located in the anterior and median parts of the mandible were capable to resist various stresses acting together (compression *vs* tension) which was helpful during feeding activity. In contrast, in the posterior and articular areas of the mandible only compression-resistant sutures have been noted. Such suture types combined with a large number of long Sharpey’s fibres may have reinforced and compensated for the relatively weak microstructure of those bones.

In summary, the histology of the mandible presented herein corroborates and significantly supplements earlier histological and computational FE investigations into the ecology of metoposaurids. Moreover, we present the first description of dermal bone microanatomy of serially sectioned specimens. Based on previous works, we know that these temnospondyls specialised both in active and ambush predation. The bone microstructure and morphology of the sutures suggest that the skull was used for lateral or bilateral biting, whereas the mandible played a role in holding down any struggling prey. Due to a lack of histological data of the mandible in early tetrapods, it is difficult to compare the results scored for *Metoposaurus krasiejowensis* with other temnospondyl taxa. However, it cannot be ruled out that in temnospondyls with an analogous morphology of the mandible (*e.g*., *Cyclotosaurus*), their general histology and biomechanical role was similar. Thus, results presented here and in previous papers on mandibular sutures in *Metoposaurus krasiejowensis*, constitute a solid basis for future histological and computational studies.

## Additional Information and Declarations

### Data Availability

The following information was supplied regarding data availability: The data are available in the Figures. The two mandible specimens, UOPB 01145 and UOPB 01027, are stored in the collections of the University of Opole (Poland), Institute of Biology, Laboratory of Palaeobiology.
